# ATF4 in proximal tubules modulates kidney function and modifies the metabolome

**DOI:** 10.1007/s00109-025-02559-4

**Published:** 2025-06-21

**Authors:** Yuling Chi, Eduardo Mere Del Aguila, Tuo Zhang, Charles D. Warren, Helen R. Hoxie, Qiuying Chen, Steven S. Gross, Jacob B. Geri, David M. Nanus, Lorraine J. Gudas

**Affiliations:** 1 Pharmacology Department, Weill Cornell Medical College of Cornell University, 1300 York Avenue, New York, NY 10065-4896 USA; 2 The Genomics Core of Cornell University, 1300 York Ave, New York, NY 10065 USA; 3 The Department of Medicine of Cornell University, 1300 York Ave, New York, NY 10065 USA; 4 Weill Cornell Medicine and Weill Cornell Graduate School of Medical Sciences of Cornell University, 1300 York Ave, New York, NY 10065 USA

**Keywords:** Activating transcription factor 4 (ATF4), Kidney proximal tubules, Transporters, Transcriptomics, Proteomics, Metabolomics

## Abstract

**Abstract:**

Activating transcription factor 4 (ATF4) is a transcription factor that mediates the response to stress at the cellular, tissue, and organism level. We deleted the gene encoding ATF4 in the proximal tubules of the mouse kidney by using a temporal and cell type-specific approach. We show that ATF4 plays a major role in regulating the transcriptome and proteome, which, in turn, influences the metabolome and kidney functions. Genome-wide transcriptomics and single-plot, solid-phase-enhanced sample preparation (SP3)-proteomics studies reveal that ATF4 deletion changes more than 30% of transcripts and, similarly, corresponding proteins in the proximal tubules. Gene Set Enrichment Analysis indicates major changes in transporters, including amino acid transporters. Metabolomic analyses show that these changes in transporters are associated with altered profiles of amino acids in the blood, kidney, and urine. Stable isotope glutamine tracing in primary tubule cells isolated from kidney cortices confirms that ATF4 regulates glutamine transport and metabolism. We suggest that even in the absence of additional stresses, such as kidney injury, ATF4 in the proximal tubules modulates both retention of specific nutrients and excretion of catabolic products like creatinine to maintain normal kidney function.

**Key messages:**

- Activating transcription factor 4 (ATF4) deletion changed more than 30% of genome-wide transcripts and corresponding proteins in the proximal tubules.
- One set of the profound changes occurred in amino acid transporters and Slc22 family transporters.
- Changes in transporters were accompanied by altered profiles of amino acids and wastes in the blood, kidney, and urine.
- ATF4 in the kidney proximal tubules plays a key role in regulating both the reabsorption of nutrients and the excretion of wastes.

**Supplementary Information:**

The online version contains supplementary material available at 10.1007/s00109-025-02559-4.

## Introduction

ATF4 (activating transcription factor 4), a transcription factor induced by various types of cellular stress, is a major mediator of the integrated stress response (ISR) [1]. The ISR is an adaptive signaling pathway activated during potentially pathological conditions, such as glucose and/or amino acid deprivation, viral infection, hypoxia, mitochondrial stress, and oncogene activation [2–4]. In normal (non-malignant) cell types, there are different outcomes of the ISR, depending in part on the level and the duration of ISR pathway activation. Low-level activation or short-duration ISR pathway activation increases pro-survival pathways, whereas apoptotic pathways can be activated when the ISR is active for longer time periods and/or at higher levels [3].

Growth factor signaling via mTORC [5] can also activate ATF4, in part to promote glutathione synthesis [6] and nonessential amino acid synthesis [7]. ATF4 targets include transcripts that encode some biosynthetic proteins, such as enzymes in the serine-glycine pathway and a subset of amino acid transporters [8, 9].

Whole body ATF4 knockout (KO) mice generally survive beyond birth, but these mice are anemic and are smaller than wild type (WT) [10]. They are also lean and show increased energy expenditure [11]. Additionally, whole body ATF4 KO mice are hypoglycemic and are resistant to diet-associated diabetes, hyperlipidemia, and hepatosteatosis; the liver and adipose tissue from these mice exhibit reduced mTOR signaling [12].

Because analysis of whole body ATF4 KO mice is complicated, tissue-specific ATF4 KO mice have also been generated and assessed. The responses to *tissue-specific knockout* of ATF4 are context dependent, varying in different tissues and with different models of cellular stress. ATF4 deletion is in some cases detrimental and in others beneficial to the tissue and organism. For instance, expression of transcripts involved in the response to oxidative stress is reduced in liver-specific ATF4 KO mice, leading to greater oxidative damage during endoplasmic reticulum (ER) stress [13, 14]. However, liver-specific ATF4 KO mice show resistance to ethanol-induced liver steatosis [14]. In skeletal muscle-specific ATF4 KO mice, less atrophy occurs in response to stress, and these mice are also protected from age-associated reductions in exercise ability, strength, and muscle mass [15, 16]. Cardiomyocyte-specific ATF4 KO mice, in contrast, show greater cardiomyopathy after aortic constriction [17]. ATF4 deletion in intestinal epithelia results in more intestinal inflammation [18]. ATF4 deletion in hemopoietic stem cells causes an aging phenotype, in part from a reduction in HIF1α signaling and an increase in mitochondrial reactive oxygen species [19].

ATF4 is highly expressed in the kidney. However, its role in the regulation of kidney functions is largely unknown. We recently demonstrated that ATF4 and its target genes are expressed at higher levels in human clear cell renal cell carcinoma than in normal kidney [20]. We also showed that a knockout of ATF4 in PTs affects kidney carcinogenesis [21]. These findings prompted us to assess the functions of ATF4 in normal kidney proximal tubule (PT) cells by comparing gene and protein expressions, metabolites, and kidney functions in mice in which ATF4 is selectively deleted in PT cells. Here, we report that ATF4 deletion in PTs changed the expression of more than 30% of the genome and proteome, including amino acid transporters and other ion transporters. Those changes were accompanied by altered metabolic profiles in the blood, kidney, and urine, and ultimately changes in renal function.

## Methods

All experiments were approved by the Institutional Animal Care and Use Committees (IACUC) of WCMC. The homozygous floxed ATF4 (ATF4^fl/fl^) mice were a gift from Dr. Christopher M. Adams [16]. We generated Ggt/CreER^TG/TG^ (designated GCER) mice in our lab [22]. We crossed ATF4^fl/fl^ with Ggt/CreER^TG/TG^ mice to generate Ggt/CreER^TG/TG^; ATF4^(fl/fl)^ (designated GCERA) mice. We injected 200 µL of tamoxifen (Cayman Chemical, #13258, MI) in cottonseed oil (Sigma, #C7767) at a concentration of 20 mg/mL into 6-week-old mice once/day for 2 consecutive days to specifically delete the ATF4 gene in PTs of the kidney, designated GCERAΔ.

Detailed experimental methods are described in Supplementary Information.

## Results

### Generation and verification of the ATF4 knockout specifically in proximal tubules

**Fig. 1:**
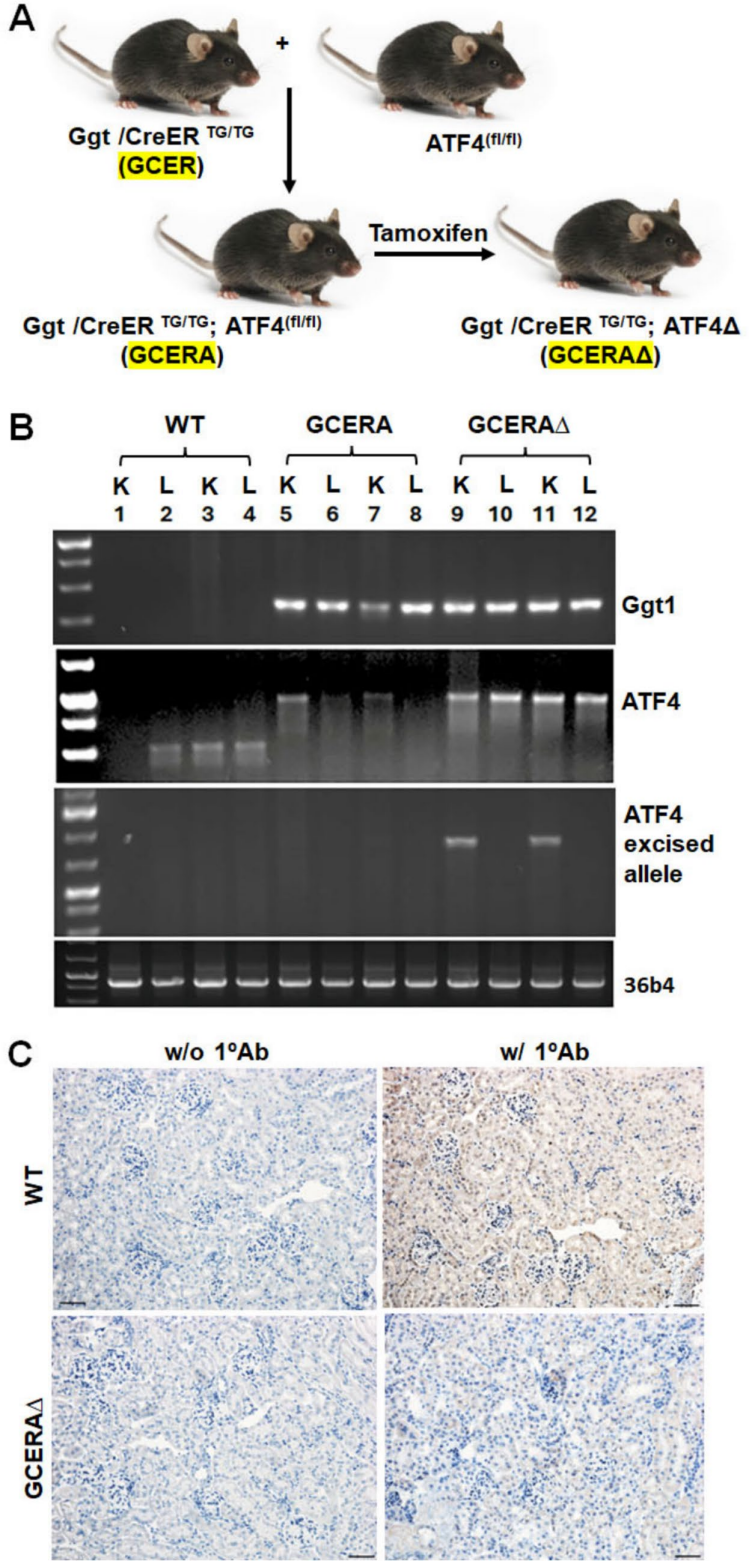
Generation and verification of the ATF4 knockout specifically in proximal tubules. **A** Generation of GgtCreER; ATF4 knockout (KO) (GCERAΔ) mice. **B** Genotyping with DNA extracted from the kidney and liver (as negative reference) and PCR as described in the “Methods” section; **C** Staining of kidney tissues with ATF4 antibody. WT: wild type; GCERA: ggtCreER; ATF4^fl/fl^; GCERAΔ: ggtCreER; ATF4^fl/fl^ after treatment with tamoxifen; K: kidney; L: liver

Gamma glutamyltransferase 1(Ggt1) is abundantly and selectively expressed in the PTs of the kidneys, particularly in PT segment 3 (Kidney Interactive Transcriptomics **(**KIT)**,** https://humphreyslab.com/SingleCell/). We engineered a murine transgenic (Tg) line with a portion of the Ggt1 promoter driving CreER [22] (GCER) for tamoxifen-inducible deletion of any floxed gene in the PTs at any desired age of mice (Fig. 1A). Crossing this Tg line with ATF4^fl/f/l^ mice resulted in the GCERA line. After tamoxifen injections on 2 consecutive days, these GCERA mice exhibited selective ATF4 deletion (GCREA∆) in the PTs (Fig. 1A, B). ATF4 protein was also expressed in PTs of WT, but not in GCREA∆ kidneys (Fig. 1C), demonstrating ATF4 specific deletion in PTs.

### Major changes in mRNAs and proteins after proximal tubule ATF4 deletion

**Fig. 2:**
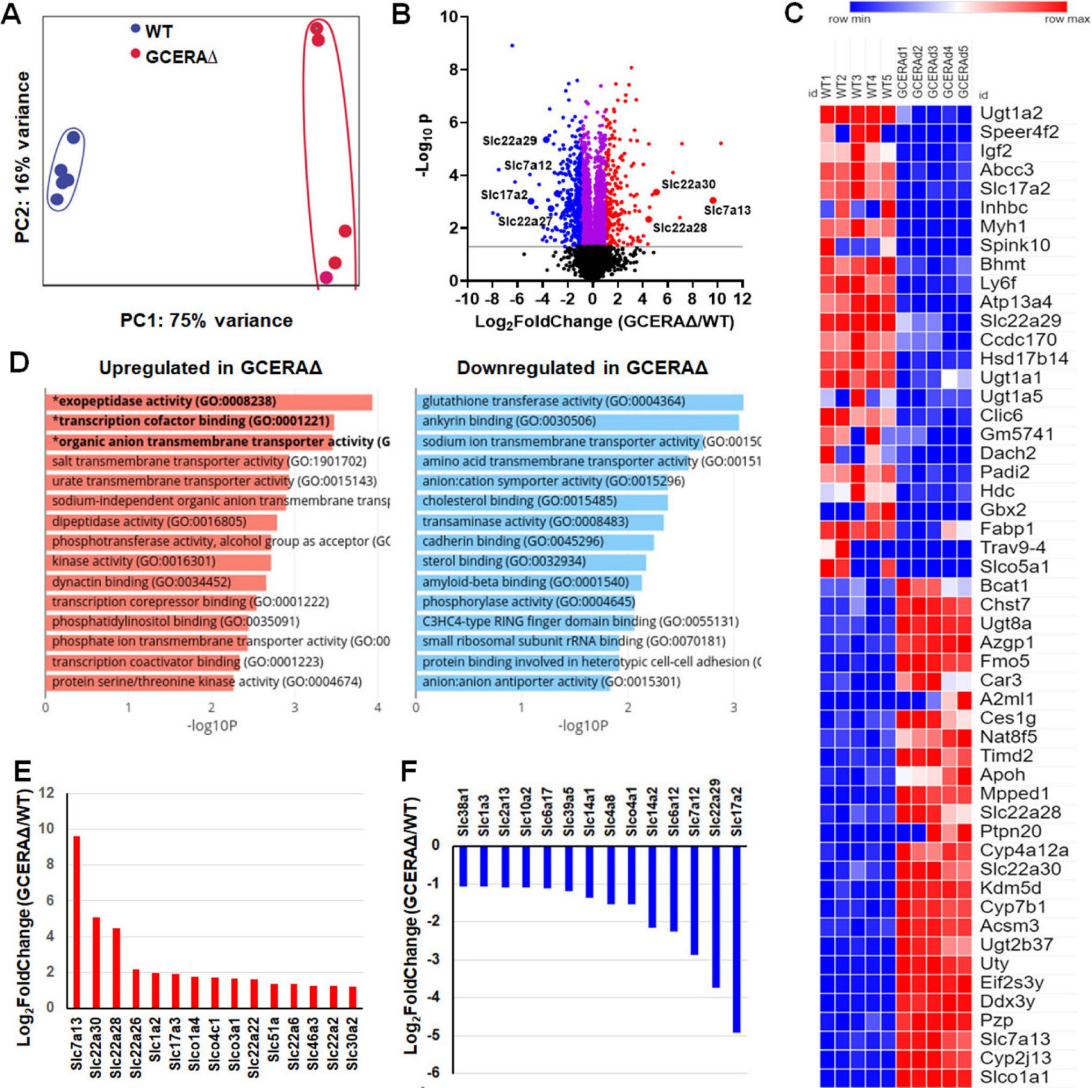
Deletion of ATF4 in proximal tubules caused genome-wide transcript changes. **A** Principal component analysis (PCA) of mRNA-seq data; **B** volcano plots of -log_10_(*p*_adj_) versus log_2_(fold change) of gene expression in GCERAΔ over WT cortices. *p*_adj_ and log_2_(fold change) were calculated using R package DESeq2. Significantly changed genes are in blue, purple, and red. Genes significantly downregulated by two-fold are in blue. Genes significantly upregulated by two-fold are in red; **C** GSEA generated heatmap of top 50 features for each phenotype; **D** pathways generated by BioJupies; **E** significantly increased Slc genes by two-fold, and **F** significantly decreased Slc genes by two-fold

Since ATF4 is a transcription factor, we first asked whether ATF4 deletion in PTs resulted in any changes in transcript levels. We extracted total RNA from kidney cortices and conducted genome-wide deep mRNA sequencing (mRNA-seq). We used kidney cortices because PT cells comprise the majority (about 60–70%) of the cortices and thus, the sequencing data will be representative of PT cell mRNAs [23]. The quality of RNA-seq data from 10 samples is summarized (Supplementary Table 2). Principal component analysis (PCA) shows that transcripts of WT and GCERAΔ samples are closely clustered and that these two groups are well separated for the majority of transcripts (Fig. 2A), indicating high reproducibility and the ability to distinguish between the transcript profiles of these two groups.

We detected 12,804 mRNAs with FPKMs of ≥ 1, 31.2% of which were significantly changed in GCERAΔ vs. WT cortices. Major mRNA changes are shown in the volcano plot (Fig. 2B). Gene Set Enrichment Analysis (GSEA) generated a heatmap of the top 50 altered mRNAs, with half of them enriched in WT and the other half enriched in GCERAΔ cortices (Fig. 2C). Gene Ontology analysis of GCERAΔ compared to WT cortices with BioJupies [24] shows that 5 out of 15 upregulated and 4 out of 15 top downregulated molecular function pathways are transporter systems (Fig. 2D). Collectively, ATF4 PT deletion caused major changes in transporter mRNAs (Fig. 2B–D). We therefore focused on transporters. Detailed analysis revealed that 288 transporter genes were detected, 50 of which were significantly increased and 75 decreased in GCERAΔ versus WT. The transcripts increased by ≥ two-fold and those decreased by ≥ two-fold in GCERAΔ compared to WT cortices are shown in Fig. 2E, F, respectively. Among transporter transcripts expressed at higher levels in GCERAΔ compared to WT cortices are Slc22a28, Slc22a30, and Slc7a13 (Fig. 2E). Among transporter transcripts with lower expression in GCERAΔ compared to WT are Slc7a12, Slc22a29, and Slc17a2 (Fig. 2F).

**Fig. 3:**
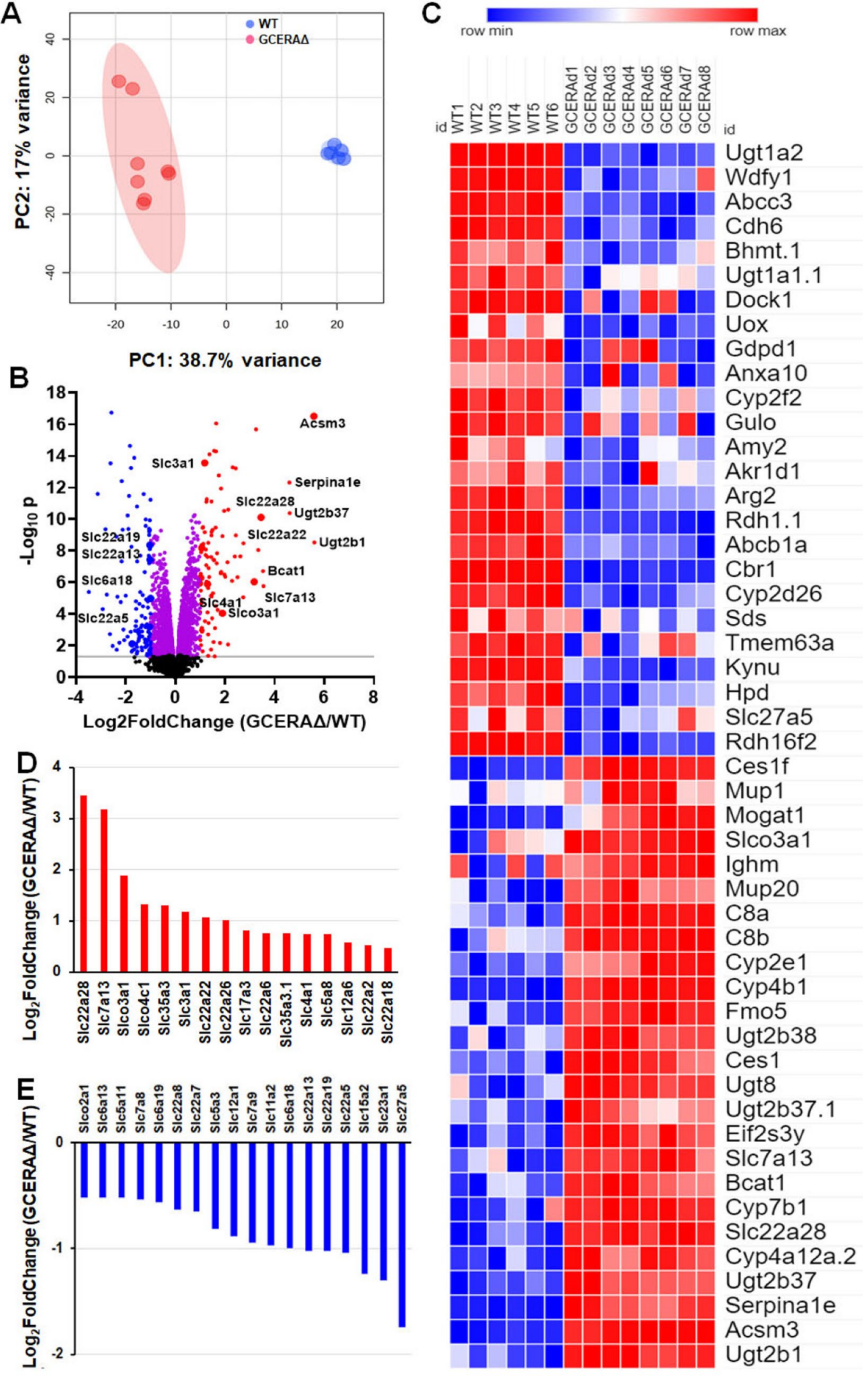
Deletion of ATF4 in proximal tubules caused proteome wide protein changes. **A** Principal component analysis (PCA) of proteomics data; **B** volcano plots of -log_10_(*p*_adj_) versus log_2_(fold change) of protein levels of GCERAΔ over WT. *p*_adj_ and log_2_(fold change) were calculated using R package DESeq2. Significantly changed proteins are in blue, purple, and red. Proteins significantly downregulated by two-fold are in blue. Proteins significantly upregulated by two-fold are in red; **C** GSEA generated heatmap of top 50 features for each phenotype; **D** significantly increased Slc proteins, and **E** significantly decreased Slc proteins

To investigate whether the ATF4-deletion associated changes in transcripts could be correlated with changes in proteins, we conducted proteome-wide proteomics studies. With filtration of the peptides ≥ 2, the total number of proteins detected in the cortices was 5471, with 2012 proteins (38.7%) significantly changed. Like the PCA of mRNAs, the PCA of proteins shows close clustering in WT and GCERAΔ cortices, respectively, and these two groups are well separated (Fig. 3A). The volcano plot (Fig. 3B) shows that transporters represent the major class of proteins that exhibit changes, also indicated as top-ranked protein enrichments in the heatmap generated by the GSEA (Fig. 3C). Detailed changes in some proteins are shown in Fig. 3D, E.

### PT ATF4 deletion affects transporters for inorganic cations/anions and amino acids/oligopeptides in the kidney cortex

**Fig. 4:**
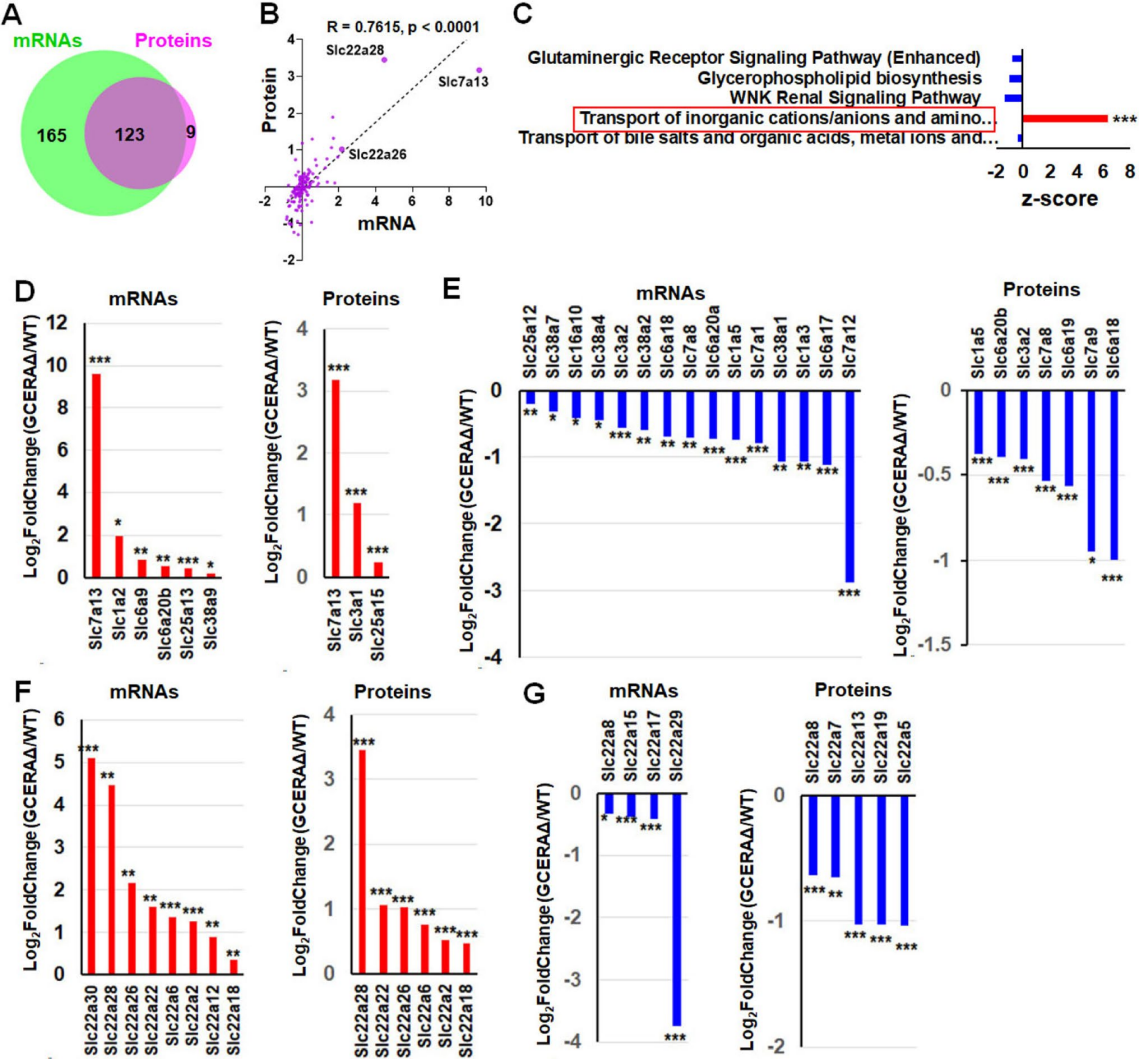
Deletion of ATF4 significantly affected amino acid transporters (AATs) and Slc22 family transporters at both mRNA and protein levels. **A** Venn diagram of all detected transporter mRNAs and proteins; **B** correlation between shared 123 transporter mRNAs and proteins; **C** pathways generated by Ingenuity Pathway Analysis (IPA) of all transporter mRNAs. Positive *Z* score is in red, negative *Z*-scores are in blue; **D** significantly increased AATs; **E** significantly decreased AATs; **F** significantly increased Slc22 family transporters; **G** significantly decreased Slc22 family transporters. *p ≤ 0.05; **p ≤ 0.01; ***p ≤ 0.0001

Both transcriptomics and proteomics studies demonstrate that the major changes caused by ATF4 deletion are in transporters. Thus, we uploaded all transporter mRNAs and proteins into the DeepVenn program [25] and performed correlation analysis. Almost all detected transporter proteins are translated from the detected mRNAs (Fig. 4A), and 80% of the shared mRNAs are positively correlated with their corresponding proteins (Fig. 4B).

Since many types of transporters are expressed in the kidney [26], we conducted Ingenuity Pathway Analysis (IPA) of all 288 transporters. The pathways for transport of inorganic cations/anions and amino acids/oligopeptides exhibited the greatest changes (Fig. 4C). Therefore, we examined these amino acid transporters (AATs) in more detail. Of 45 detected AATs, Slc7a13 was the only one that showed increases at both mRNA and protein levels in GCERAΔ compared to WT (Fig. 4D). Slc7a13 (formerly AGT-1) is localized on the apical side of the PT [27]; Slc7a13 transports glutamate (Glu), aspartate (Asp), and cystine from urine to blood [28]. Compared to the number of mRNAs/proteins increased by the ATF4 deletion, many more AAT mRNAs/proteins were decreased in GCERAΔ compared to WT (Fig. 4E). The mRNA (Slc7a12) showing the greatest decrease was not detected in our proteomics analysis.

Additionally, some members of the Slc22 family were changed by ATF4 deletion. Twenty-one Slc22 family genes were detected. Six of eight transcripts increased in GCERAΔ compared to WT were also increased at the protein level in GCERAΔ compared to WT (Fig. 4F). One of these, Slc22a2, is located on the PT basolateral side [29]. One of Slc22a2’s substrates is creatinine [30], and another substrate, notably, is cisplatin [31]. Increased expression of Slc22a2 in the ATF4 knockout PTs could potentially lead to transport of more creatinine from the blood to the urine, increasing urine creatinine. Among the Slc22 family transcripts decreased in GCERAΔ compared to WT, only Slc22a8 showed lower mRNA and protein levels (Fig. 4G).

### PT ATF4 deletion alters metabolite profiles in serum, kidney, and urine

**Fig. 5:**
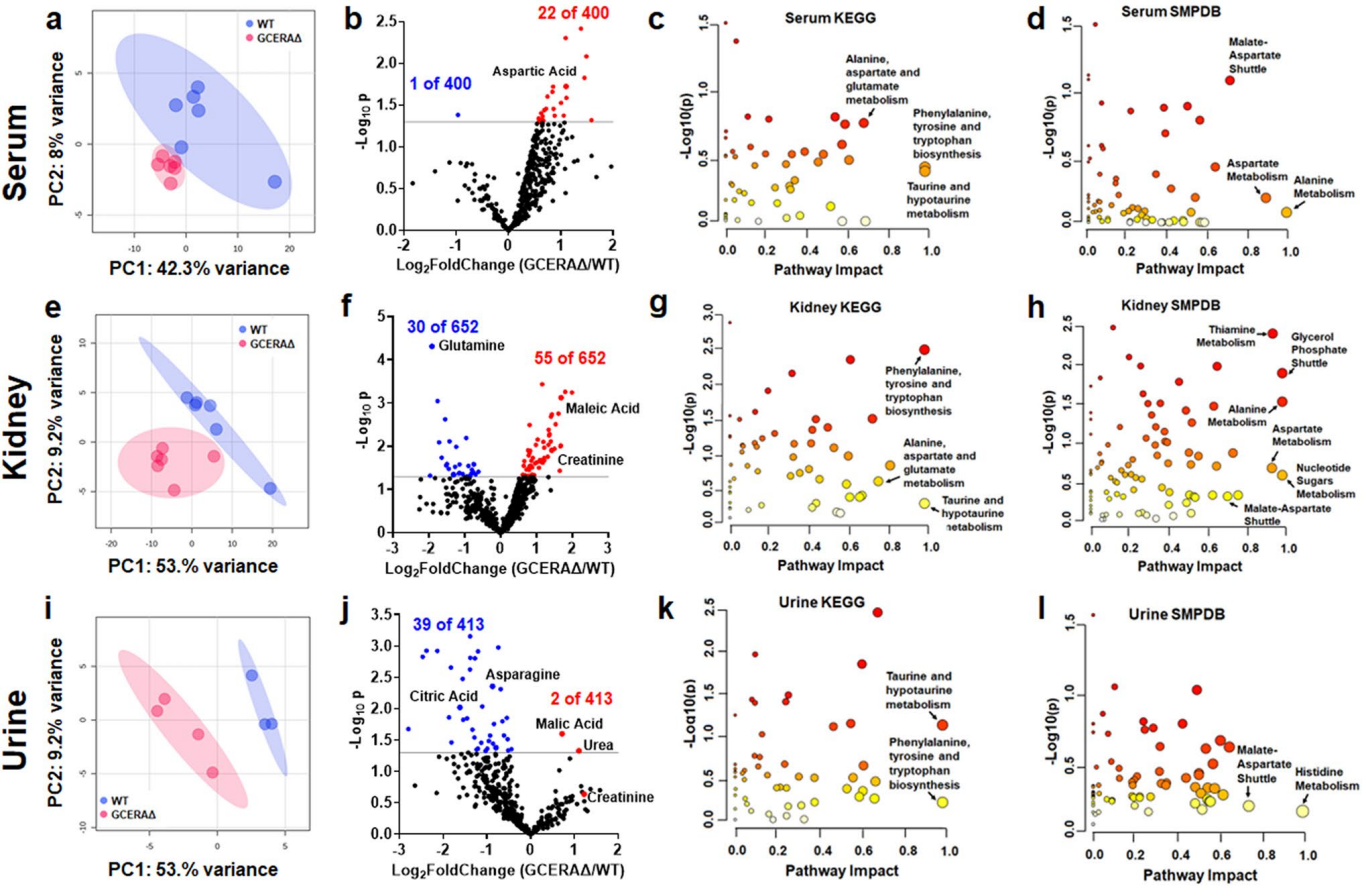
Deletion of ATF4 in proximal tubules caused changes in metabolite profiles in the serum, kidney, and urine. **A**, **E**, **I** Principal component analysis (PCA) of metabolites in the serum, kidney, and urine; **B**, **F**, **J** volcano plots of -log_10_(*p*_adj_) versus log_2_(fold change) of metabolites of GCERAΔ over WT. Significantly reduced metabolites are in blue; significantly increased metabolites are in red. **C**, **G**, **K** Kyoto Encyclopedia of Genes and Genomes (KEGG) pathways of metabolites in the serum, kidney, and urine generated by MetaboAnalyst; **D**, **H**, **L** Small Molecule Pathway database (SMPDB) pathways of metabolites in the serum, kidney, and urine generated by MetaboAnalyst

Since ATF4 deletion in PTs changed large numbers of proteins, some of which carry out metabolic reactions, we hypothesized that the PT ATF4 deletion could alter renal metabolism. We took an untargeted approach, profiling all metabolites in all three compartments, the blood/serum, kidney, and urine, by LC/MS. PCA shows that the metabolites in serum are closely clustered in WT vs. GCERAΔ cortices and that these two groups are separated by principal component-1 (PC-1) of 42.3% of metabolites (Fig. 5). Greater separations between GCERAΔ and WT are found in kidney (Fig. 5E) and urine (Fig. 5I). These PCAs demonstrate that there are metabolite differences between WT and GCERAΔ in all three compartments.

The serum volcano plot shows that all significantly changed metabolites (23/400), except one, are increased in GCERAΔ compared to WT (Fig. 5B). One of the metabolites increased in GCERAΔ cortices is Asp. More metabolites (85/652) were altered in the kidneys of GCERAΔ vs WT than in serum or urine (Fig. 5F). Contrary to the changes in serum, all significantly changed metabolites (41/413) in urine are reduced in GCERAΔ compared to WT, except for malic acid and urea (Fig. 5J). Urine creatinine is higher in GCERAΔ compared to WT. Overall, ATF4 deletion in PTs was associated with increased concentrations of metabolites in serum and decreased concentrations of metabolites in urine (Fig. 5B, J), suggesting that ATF4 deletion causes enhanced uptake of metabolites from urine to serum as well as increased excretion of wastes such as urea and creatinine.

Pathway analysis using the MetaboAnalyst KEGG database [32] shows that ATF4 deletion has a significant impact on many metabolic pathways. Among those are Phe-Tyr-Trp synthesis and Ala-Asp-Glu metabolism (Fig. 5C, G, K). Pathway analysis based on the Small Molecule Pathway Database (SMPDB) [33] shows similar impacts on pathways such as Ala metabolism, Asp metabolism, and the malate-Asp shuttle in GCERAΔ vs WT (Fig. 5D, I, L).

**Fig. 6:**
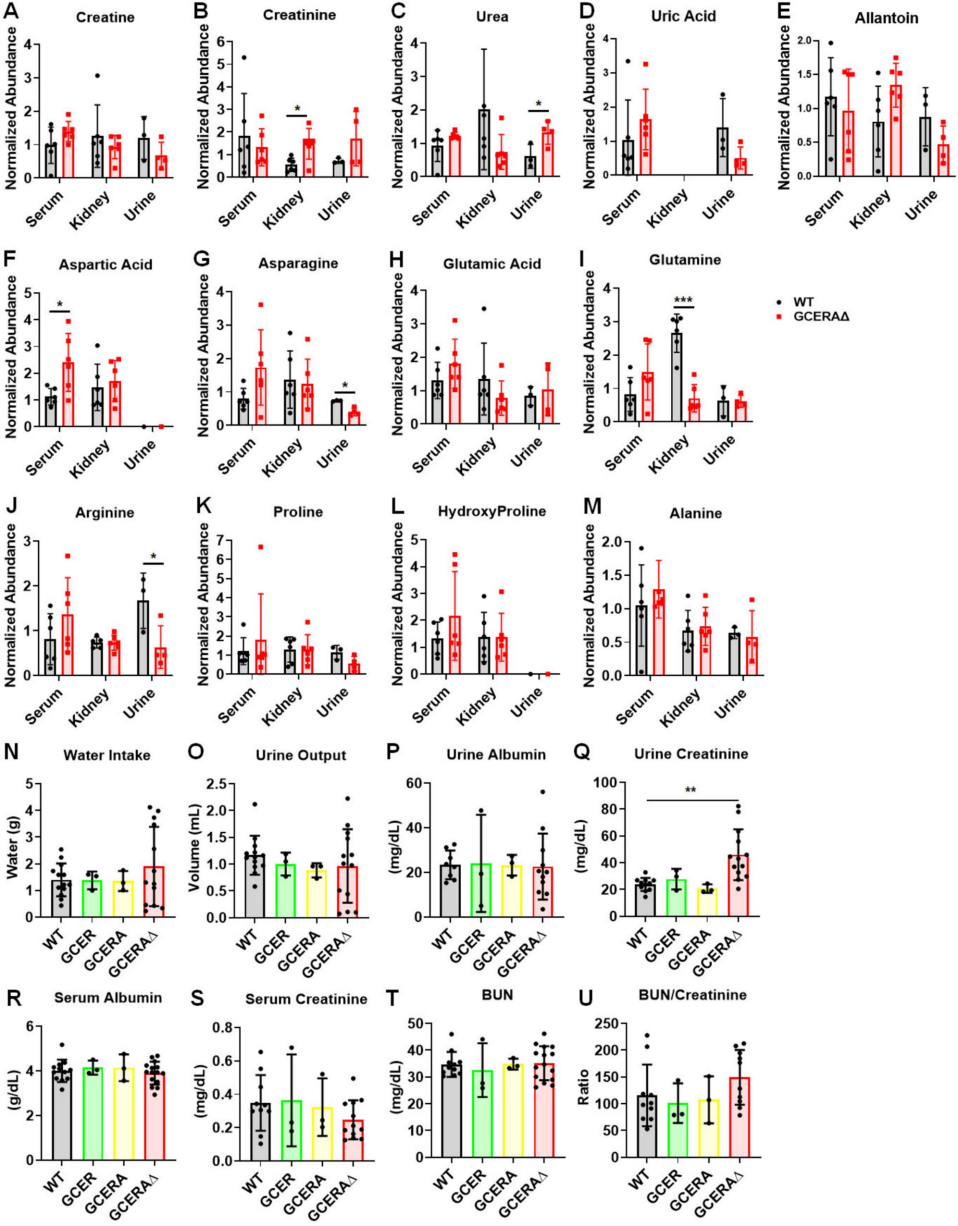
Deletion of ATF4 in proximal tubules affects excretion of wastes and reabsorption of nutrients. **A**–**M**, metabolites in the serum, kidney, and urine analyzed by metabolomics studies. **N**–**U**, markers of renal functions measured by spectrometry. Transporters in red and blue are increased and reduced in GCERAΔ compared to WT, respectively. *p ≤ 0.05; **p ≤ 0.01; ***p ≤ 0.0001

PT ATF4 deletion increased creatine, an energy source for muscle partially supplied by the kidney, in serum from GCERAΔ mice, but decreased creatine in urine (Fig. 6A). In contrast, creatinine, a chemical waste product of creatine, was decreased in serum but was significantly increased in the kidneys and urine of GCERAΔ compared to WT (Fig. 6B). Urea, excreted in the urine, is a breakdown product of proteins and/or amino acids [20], whereas uric acid is primarily a breakdown product of nucleotides such as DNA or RNA [34]. In mouse, uric acid is oxidized to allantoin and excreted in urine [35], which is different from what occurs in humans. ATF4 deletion in PTs caused increased excretion of waste products from proteins and/or amino acids (Fig. 6C) and a trend toward a reduction in waste products from nucleotides (Fig. 6D, E).

The kidney is one of the tissues in which the highest consumption of glutamine (Gln) occurs [36]. Gln is the most abundant, naturally occurring non-essential amino acid [36]. Gln is essential for many cellular functions, including the synthesis of nucleotides and non-essential amino acids [37]. ATF4 deletion in PTs reduced Gln levels in the kidney by almost four-fold and increased Gln in serum by two-fold (Fig. 6I). A similar trend occurred for Glu (Fig. 6H). Glu can be converted to Ala and Asp, which are precursors for the synthesis of several other amino acids [38]. Increased Glu probably contributes to the increased Asp in GCERAΔ blood compared to WT (Fig. 6F). Asp can also be produced by hydrolysis of asparagine, which is also increased in serum but decreased in the urine of GCERAΔ compared to WT (Fig. 6G). PT ATF4 deletion also affected arginine levels (Fig. 6J), but not those of proline or Ala (Fig. 6K–M). Additional amino acids affected by PT ATF4 deletion are shown in Supplementary Fig. 1.

### PT ATF4 deletion affects glutamine metabolism in primary tubule cells

**Fig. 7:**
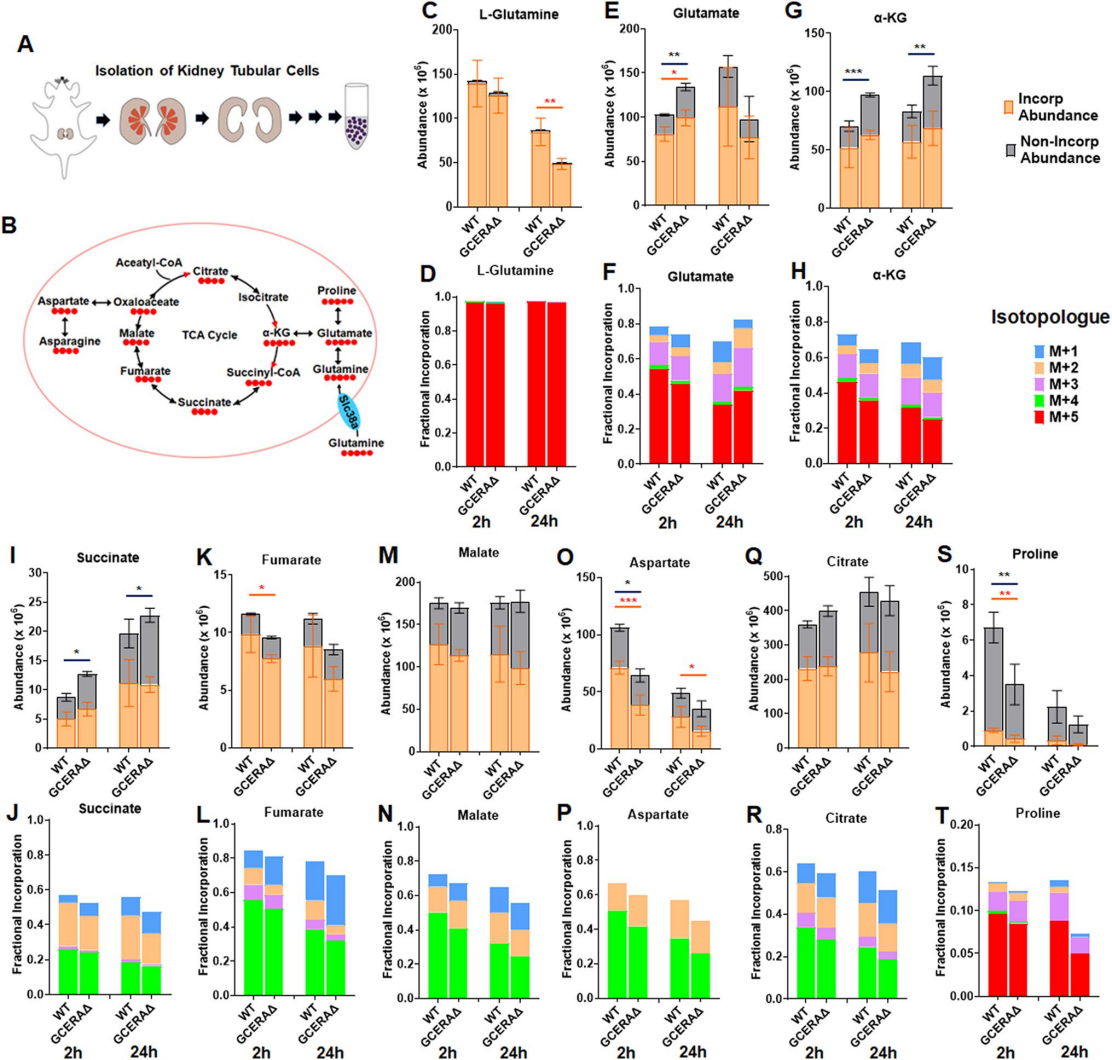
Deletion of ATF4 in proximal tubules affects glutamine metabolism in primary tubular cells. **A**, diagram of isolation of primary tubule cells from kidney cortices; **B**, diagram of uptake and metabolism of [U^13^C_5_]glutamine; **C**–**T**, abundances of metabolites incorporated or non-incorporated with ^13^C isotopes and their corresponding fractional incorporations at 2 h and 24 h after cells were fed with [U^13^C_5_]glutamine. Transporters in blue in **B** are reduced in GCERAΔ compared to WT. α-KG, α-ketoglutarate. *p ≤ 0.05; **p ≤ 0.01; ***p ≤ 0.0001. Orange stars indicate significance for comparisons of abundances incorporated with ^13^C isotopes between GCERAΔ and WT; black stars indicate significance for comparisons of abundances non-incorporated with ^13^C isotopes between GCERAΔ and WT

To confirm the effects of PT ATF4 deletion on nutrient metabolism, we isolated primary tubule cells from cortices of GCERAΔ and WT mice (Fig. 7A) and conducted [U^13^C_5_]glutamine tracing in those cells. [U^13^C_5_]glutamine uptake and metabolism were stopped at 2 h and 24 h, respectively, and intracellular metabolites were analyzed by LC/MS. Almost all detected intracellular glutamine contained ^13^C (Fig. 7C), and all five carbons were ^13^C (Fig. 7D). However, [U^13^C_5_]glutamine level was much lower in GCERAΔ than in WT cells, most likely resulting from lower expression of glutamine transporters (Slc38a1,2,4,7) (Fig. 4E). Glutamine was converted to glutamate, which could be metabolized to different metabolites (Fig. 6B). One of the major pathways is metabolism to α-ketoglutarate (α-KG), which subsequently goes through the TCA cycle. While there is more α-KG with ^13^C (Fig. 7G) in GCERAΔ cells, the amount of α-KG with 5 ^13^Cs was lower in GCERAΔ cells (Fig. 7H), i.e., α-KG directly generated from glutamine was lower in GCERAΔ than WT. Alpha-KG was subsequently metabolized to 4-C metabolites in the TCA cycle. All 4-C metabolites were lower in GCERAΔ than WT cells (Fig. 7I–N, Q,R). Aspartate converted from oxaloacetate was also lower in GCERAΔ than WT cells (Fig. 7O, P). Glutamate can be converted to proline. Though the contribution of incorporated proline to the total proline is minimal, the incorporated proline from the glutamine tracer was much lower in GCERAΔ than WT (Fig. 7S, T).

Glutamine transporters (Slc38a1,2,4) are located on the basolateral sides of proximal tubules [28], taking glutamine from the blood into cells. The lower intracellular abundance of glutamine metabolites suggests that fewer nutrients are inside PT cells to be filtered out and that more nutrients are retained in the circulation in the GCERAΔ than WT. These tracing results are consistent with metabolomics data (Fig. 6F–K).

### ATF4 deletion affects kidney function

To assess if the effects of ATF4 PT deletion are manifested at the physiological level, we measured kidney functions by placing mice in metabolic cages and recording their water intake and urine output. We collected their urine and blood for measurements of albumin and creatinine in both serum and urine, and of blood urea nitrogen (BUN) in serum. We also included two additional groups (GCER and GCERA) as controls to assess if the transgenes themselves exerted any effects on renal functions. As anticipated, there were no significant differences among the WT, GCER, and GCERA cohorts for all measurements (Fig. 7). While we observed no differences in water intake (Fig. 7N) and urine output (Fig. 7P), urinary creatinine was doubled in GCERAΔ compared to WT (Fig. 7Q). Conversely, serum creatinine was lower in GCERAΔ vs WT (Fig. 7S). These changes in urinary and serum creatinine are consistent with those obtained from metabolomics studies (Fig. 7B). While slightly lower urine volumes in the GCERAΔ mice could contribute to their higher urine creatinine levels, increased export of creatinine, likely resulting from higher expression of the creatinine transporter (Slc22a2) [30] (Fig. 4F) on the basolateral side of the PT [29, 39], could explain the higher urine creatinine in GCERAΔ compared to WT. We observed no differences in albumin in either the urine or blood among these four groups (Fig. 7P, R). PT ATF4 deletion did not cause changes in BUN (Fig. 7T) nor in the BUN to creatinine ratio (Fig. 7U), which is also consistent with the serum urea levels measured from the metabolomics studies (Fig. 7C).

## Discussion

ATF4 is a transcription factor responding to various types of stress [1]. Utilizing multi-omics technology, we demonstrate that under basal conditions, ATF4 deletion in kidney PTs results in major changes in the genome-wide transcriptome and proteome. The changes in mRNAs and proteins are associated with altered metabolic profiles in the urine, kidney, and blood, and renal physiology (Fig. 6N–U).

Many roles of ATF4 in the regulation of development, lipid metabolism, and thermogenesis have been elucidated by using whole body ATF4 knockout mice [11, 13]. Mice with ATF4 specifically deleted in various tissues, including the muscle [3], liver [12], heart [17], intestine [18], and brown adipose tissue [40], have also been generated to analyze ATF4’s actions in regulating muscle aging, heart health, gluconeogenesis, and fatty acid synthesis under basal and or stressed conditions. However, this is the first report focusing on the regulation of kidney function under basal conditions by ATF4 in PTs.

One set of positively correlated genes and proteins that is affected by ATF4 deletion is the solute carriers (Slcs) (Fig. 4B), which are the largest family of transmembrane transporters carrying out major physiological functions in the kidney. Slcs are classified into several families. We found that the most profound changes caused by ATF4 deletion were in the AAT family. There are a few prior reports about ATF4 regulation of AATs. In cultured mouse embryonic fibroblasts, Harding et al. [8] showed that ATF4 deletion reduced Slc7a5 and Slc3a2 transcripts. In skeletal muscle cells, an amino acid response element near the Slc38a9 gene was shown to be an ATF4-binding site [41]. Promoters of some genes with functions in amino acid transport (Slc7a11, Slc38a1, and Slc43a1) were occupied by ATF4 in DLD-1 MycER tumor cells [42]. The promoters of AATs (Slc6a9, Slc7a1, Slc7a5, Slc36a1, Slc36a4, Slc38a1, Slc38a3) were associated with ATF4 in cultured HCT116 cells [43]. These prior studies were all performed in cultured cells rather than in mice, however. Thus far, there is only one report showing a positive correlation between Slc1a5 and ATF4 mRNAs in ileal epithelia of mice with ATF4 deleted in intestinal epithelial cells [18].

**Fig. 8:**
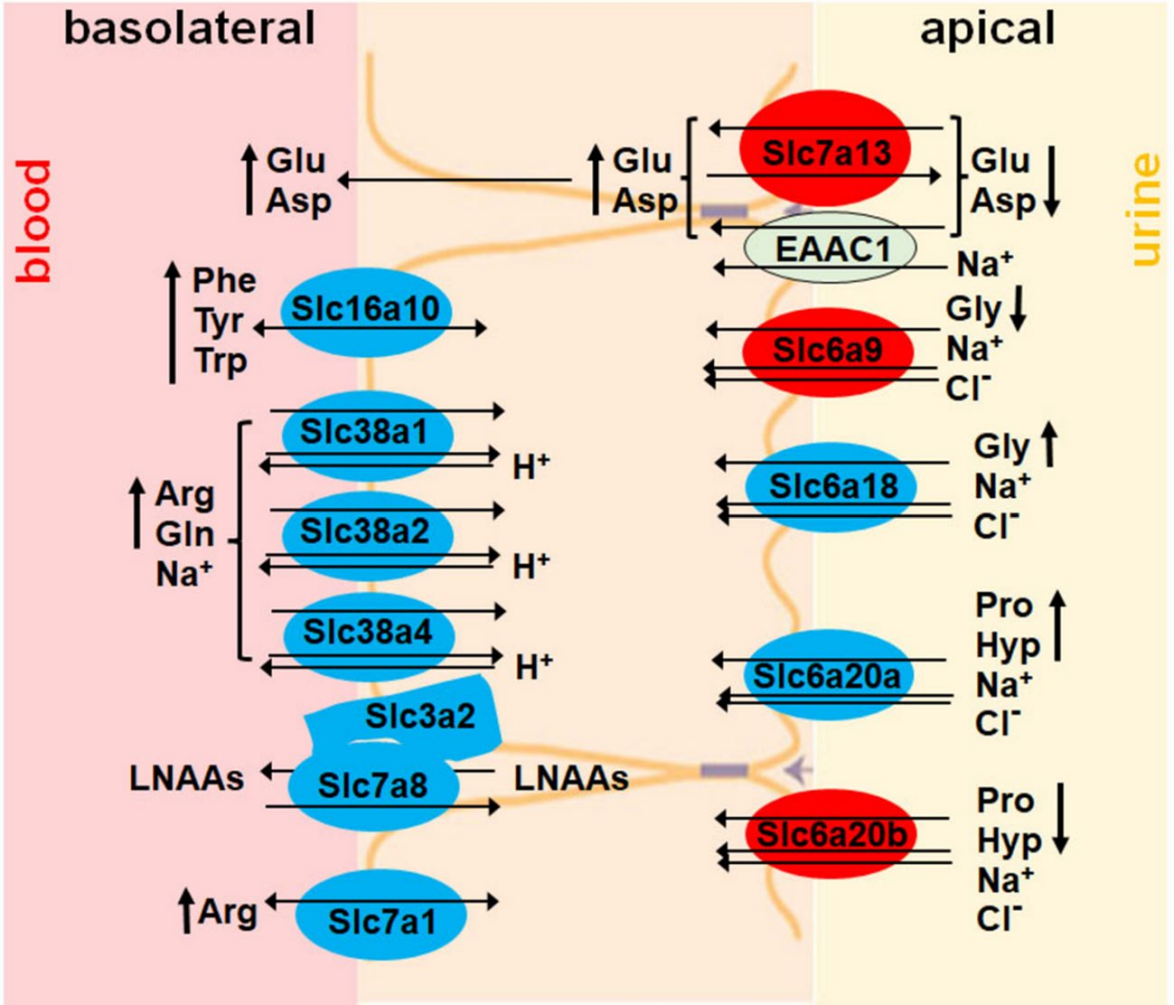
Proposed model of metabolite (mainly amino acids) changes resulted from the expression changes in transporters (mainly amino acid transporters) in kidneys of GCERAΔ mice. Transporters in red and blue are increased and reduced in GCERAΔ compared to WT, respectively

In mice under basal conditions, using untargeted, multi-omics, we identified another AAT, Slc7a13, regulated by ATF4 (Fig. 4B, D, E). A member of the AAT family, Slc7a13 resides on the apical side of PTs and re-uptakes Glu and Asp from urine (Fig. 8) [27]. We hypothesize that increased Slc7a13 mRNA and protein levels in GCERAΔ compared to WT cortices (Fig. 4D) may result in increased Glu and Asp in the blood of GCERAΔ compared to WT (Fig. 6F–I). On the apical side, besides Slc7a13, expression of two AATs was reduced, and two AATs were increased (Fig. 8).

Additional AATs reduced by ATF4 deletion (Fig. 4D, E) are localized on the basolateral side, among which are several Slc38 family members (Fig. 8) [28]. The main substrate of the Slc38 family is glutamine [44]. In response to starvation, ATF4 activates transcription of Slc38a2 in HepG2 and Hela cells [44, 45]. Here, we show that ATF4 deletion reduces Slc38 family expression (Fig. 4D, E), and thereby likely reduces the loss of glutamine and its metabolites (Figs. 6F–K and 7C–V), retaining more energy substrates [44]. Overall, the changes in AATs located on the basolateral and apical sides of ATF4 KO PT cells are associated with the retention of several AAs critical for building proteins and for cellular and physiological functions.

ORCTL3 (Slc22a13) overexpression was reported to increase ATF4 protein in HeLa, HEK293T, and HUVEC cells [46], but to date, there are no reports of ATF4 regulation of Slc22 family transporters. Here, we report that ATF4 regulates transporters of the Slc22 family. Within the Slc22 family, there are six subfamilies, including the organic anion transporter (OAT) and organic cation transporter (OCT) subfamilies [39, 47]. The changes in Slc22 transporters from the ATF4 deletion are mostly in OATs, although two OCTs (Slc22a2 and Slc22a15) are also changed (Fig. 4F, G). Overall, more OATs were increased than decreased in GCERAΔ vs. WT. Slc22a2, Slc22a7, and Slc22a8 reside on the basolateral side of PT cells and transport creatinine from blood to urine [39]. ATF4 deletion increased these three transporters at both the mRNA and protein levels, potentially explaining the increased excretion of creatinine (Figs. 6B, Q and 7).

Besides transporters, another set of genes/proteins, apolipoproteins, was significantly altered by ATF4 deletion (Supplementary Fig. 2). Apolipoproteins, such as Apol1 [48], play important roles in renal functions and kidney diseases [49]. Thus far, there are no reports about the regulation of apolipoproteins by ATF4 in the kidney. Thus, our data (Supplementary Fig. 2) could open new avenues of investigation concerning how ATF4 regulates apolipoproteins. We also discovered that ATF4 deletion affects the expression of many ribosome genes/proteins. Some of the changes are similar to those seen in hematopoietic stem cells [50].

As ATF4 is a transcription factor, some of its known target genes [20], such as ATF5 and Eif4ebp1, showed reduced expression as a result of ATF4 deletion (Supplementary Fig. 3), suggesting that under basal conditions, without additional stress, ATF4 plays a role in the modulation of these genes, their downstream signaling pathways, and the corresponding cell physiology. Some other related genes, including ATF6 and Ddit3, are ER stress markers. The levels of these transcripts were not affected by ATF4 deletion (Supplementary Fig. 3), suggesting that ATF4 deletion under basal conditions does not provoke ER stress.

In summary, we provide robust genome-wide transcriptome and proteome data and untargeted metabolome data in all three compartments (blood, kidney cortices, and urine). Integrating data from these multi-omics studies enabled us to demonstrate novel functions for ATF4 in the kidney. One of the profound changes from our transcriptomics and proteomics data sets is changes in expression of many transporters, accompanied by altered metabolic profiles in all three compartments. These data are of great value for further investigation into the functions of ATF4 in the regulation of renal molecular signaling and physiological functions.

## Supplementary Information

Below is the link to the electronic supplementary material.

Supplementary file1 (PDF 569 KB)

## Data Availability

Our mRNA-seq data are deposited into Gene Expression Omnibus (GEO), accession # GSE291049. Our proteomics data are deposited into MassIVE, accession # MSV000097819.
