# Supplementary material for: ATF4 in proximal tubules modulates kidney function and modifies the metabolome
Source: J Mol Med (Berl). 2025 Jun 21;103(8):989–1003. doi: 10.1007/s00109-025-02559-4 (PMC12343659; doi:10.1007/s00109-025-02559-4)
Supplement: Supplementary file 1 — Supplementary file1 (PDF 569 KB) [file 109_2025_2559_MOESM1_ESM.pdf]

Materials and Methods

Mice and treatments. All animal experiments and protocols were approved by the Institutional Animal Care and Use Committees (IACUC) of the WCMC. The homozygous floxed ATF4 (ATF4 fl/fl) mice were a gift from Dr. Christopher M. Adams at the Mayo Clinic [1]. We generated Ggt/CreER$^{TG/TG}$ (designated GCER) mice in our lab [2]. We then crossed ATF4$^{fl/fl}$ with Ggt/CreER $^{TG/TG}$ mice to generate Ggt /CreER $^{TG/TG}$; ATF4$^{(fl/fl)}$ (designated GCERA) mice. We injected 200 µL of tamoxifen (Cayman Chemical Company, #13258, MI) in Cottonseed oil (Sigma, C7767) at a concentration of 20 mg/mL into 6-week-old mice once per day for two consecutive days to specifically delete the ATF4 gene in PTs of the kidney, designated GCERAΔ. One month after the first injection, mice were placed in metabolic cages and fasted for 16 h. Their water intake and urine output were measured. We collected their urines for measurements of urine creatine, albumin and other metabolites. Mice were then sacrificed. Blood was collected by heart puncture and tissues harvested. Half of one kidney was placed in RNALater for total RNA extraction. The other half of the same kidney was fixed for immunohistochemstry (IHC). One sixth of the other kidney was used for DNA extraction. The rest was snap frozen in liquid N_2 for protein extraction, profiling of metabolites, and other studies.

Genotyping. The transgenic mice were genotyped using these primers (**Supplementary Table S1**). The PCR for Cre was performed with an initial denaturation step at 95°C for 10 min, followed by 34 cycles consisting of denaturation at 95°C for 1 min, annealing at 60°C for 2 min, elongation at 72°C for 1 min, and a final extension at 72°C for 10 min. For the ATF4 floxed (fl/fl) gene, PCR was performed with an initial denaturation step at 95°C for 3 min, followed by 30 cycles at 95°C for 30 sec, 57°C for 30 sec, 72°C for 90 sec, and a final extension at 72°C for

5 min. To assess the knockout induced by tamoxifen, the primers which yielded the excised genomic DNA product were used with an initial denaturation step at 95°C for 5 min, followed by 35 cycles at 95°C for 30 sec, 60°C for 30 sec, 72°C for 45 sec, and a final extension at 72°C for 5 min.

We used primers for the amplification of the transcription factor HOXA1 DNA as a positive control for DNA quality. PCR was performed at 94°C for 10 min, followed by 39 cycles consisting of 94°C for 1 min, 60°C for 2 min, and 72°C for 1 min, with a final extension at 72°C for 10 min.

Immunostaining. We fixed kidney samples in 4% paraformaldehyde buffer (pH 7.4) and embedded them in paraffin blocks. Next, we stained 5 μm sections. The slides were washed with xylene for 5 min twice, 100% ethanol for 2 min twice, 95% ethanol for 2 min twice, 70% ethanol for 2 min twice, and with dH_2O for 2 min twice. We incubated the slides using 15 mL antigen unmasking solution (Vector antigen Unmasking solution Citrate-base, pH 6 Cat H-3300 or Tris-Based, pH9 cat H3300) in 1.6 L of dH_20 for 4 min at 176°C in a pressure cooker. The slides were then incubated with 3% hydrogen peroxide in methanol for 15 min to quench endogenous peroxidase activity, followed by incubation for 10 min in PBS-T and 10 min in PBS. The slides were blocked with 10% normal goat serum (Vector Laboratories, S-1000) for 20 min at room temperature. Slides were then incubated with primary antibody (Ab) for ATF4 (Cell Signaling, 11815S) at a dilution of 1:50 for 1 h at room temperature and then overnight at 4°C. To assess non-specific staining, we included a negative control slide incubated without primary antibodies. After incubation with primary antibody, we washed slides 3 times in PBS, 10 min each wash, and then we incubated slides with 1x goat anti-rabbit IgG secondary antibody, poly HRP

conjugate (Invitrogen, B40962, OR) and MOM immunodetection kit (Vector Laboratories, CA) for 1 h at 22°C. After washes with PBS, the slides were incubated with 3,3'-diaminobenzidine substrate (Vector Laboratories), according to the manufacturer's instructions, counterstained with hematoxylin (Poly Scientific R&D, Bay Shore, NY), and mounted for image acquisition with a Nikon TE2000 inverted fluorescence microscope.

RNA extraction and mRNA-Seq. Half of a kidney was reserved in RNALater when mice were sacrificed. Those tissues were kept at 4ºC for 24 h and then moved to -80ºC for storage. Cortices of kidneys from 3 - 4 mice from each of the cohorts were dissected for mRNA-seq. Five -10 mg of each cortex section was transferred into a tube containing beads (Benchmark Scientific Prefilled Tubes for Homogenizer, D1032-15) and a 500 μL mixture of RLT (Qiagen, 74134) and β-mercaptoethanol. Tissues were homogenized twice for 30 seconds each with a Bead Bug homogenizer (Benchmark Scientific, Fort Lauderdale, FL). After homogenization, total RNA was extracted by following the protocol from the kit.

Total RNA was submitted to the WCM Genomics Resources Core. Quality control (QC) was run for each sample. Samples with RNA integrity number (RIN) $\geq$ 9.0 were selected and transcribed to cDNAs. Libraries (40,000,000 fragments / library) were constructed and fed into a NovaSeq 6000 for sequencing with pair-end 51 bps. The lane summary of all samples is shown in Supplementary Table 2. Percentages of all samples with >= Q30 bases are greater than 95%.

The raw sequencing reads in BCL format were processed through Illumina bcl2fastq v2.20 for demultiplexing and FASTQ conversion. Following adaptor and low-quality base trimming with Cutadapt v3.5, the reads were mapped to the GRCm38 mouse reference genome using STAR v2.7.9a [3]. Read counts per gene were extracted using HTSeq-count v0.13.5 [4].

Fragments per kilobase of transcript per million mapped reads (FPKMs) were calculated to normalize read counts. Genes with FPKM ≥ 1 were considered present and were selected for differential expression analysis between two conditions/groups. Gene differential expression analysis was performed using the DESeq2 package v1.38.3 [5]. Pathway enrichment analysis was performed with the R package clusterProfiler v4.6.2 [6], and the results were visualized using the R package ggplot2 v3.4.1.

Genome-wide Proteomics Assays. Ten-15 mg of previously frozen cortex section was transferred into a tube containing beads (Benchmark Scientific Prefilled Tubes for Homogenizer, D1032-15) and 250 μL of 0.1% (mg/mL) SDS / 1X RIPA buffer (10X RIPA from Sigma, 20-188). Tissues were homogenized twice, for 30 seconds each, with a Bead Bug homogenizer (Benchmark Scientific, Fort Lauderdale, FL). After homogenization, supernatant was transferred to another tube. Protein concentration in the supernatant was measured by using the Pierce™ BCA Protein Assay Kit - Reducing Agent Compatible (Thermo Fisher, 23250).

Five μg of protein from each sample in 30 μL 0.1% (mg/mL) SDS / 1X RIPA buffer was loaded to wells of a 96-well plate, with two wells per sample as duplicate. 1.5 μL of 200 mM DTT was added to each well, and the plate was incubated at 65 ºC for 15 min. After cooling to room temperature, 1.5 μL of 400 mM iodoacetamide (74 mg/mL in ddH_2O, final concentration: 20 mM) was added and incubated at room temperature for 30 minutes. 1.5 μL of SP3 beads (1:1 mixture of Cytiva, 65152105050250 and 45152105050250) suspended in water was then added to each sample, the samples mixed thoroughly, 40 μL of acetonitrile added, and the mixture again mixed thoroughly and allowed to stand undisturbed for 8 minutes to precipitate protein on bead surfaces. Next, the beads were pelleted using a magnetic rack, the supernatant removed, and

the beads washed 2x with 250 µL of acetonitrile and 1x with 80% ethanol / water. Beads were then transferred to a new 0.2 mL PCR plate, suspended in 40 µl of 50 mM ammonium bicarbonate containing 0.1 µg of Pierce MS-grade trypsin (90058), capped, and incubated at 37ºC overnight with continuous inversion. Beads were pelleted using a magnetic rack, and the supernatant transferred to a new plate for LC/MS/MS analysis.

MS proteomic measurements were performed using a Bruker timsTOF Pro 2 MS / nanoElute 2 nanoflow UHPLC system. Five µL of tryptic digest was analyzed for each sample, with chromatography via a two-column C18 separation method (0-35% MeCN/H_2O, 0.1% formic acid in a 20-minute gradient at 500 µL/min; trap column: Thermo PepMap Neo Trap Cartridge (174500), separation column: Bruker PepSep 10 (1893472)). MS analysis was performed in DIA mode, using a default vendor-defined instrument method (DIA short gradient) without alteration. Data was analyzed using DIANN 1.8 using an in-silico generated library, allowing for one missed cleavage, cysteine carbamidomethylation, and methionine oxidation. MS1/MS2 accuracy was set to 12.5 ppm. Protein quantification inference was performed by gene. Two-pass neural network classification was used, and cross-run normalization was disabled. High precision quantification was used. Protein quantities were normalized using variance stabilization normalization (VSN) with default settings in the MSnbase R package.

Gene or protein enrichment analysis was performed with Gene Set Enrichment Analysis (GSEA), a computational method developed by UC San Diego [7]. Using the Hallmark database [8], heatmaps of the top 25 genes or proteins enriched in WT and GCERAΔ cortices were generated based on the top 50 genes or proteins enriched in WT and GCERAΔ kidney cortices.

Metabolomics. Total metabolites were extracted from snap frozen kidney tissues using -70ºC

80:20 methanol:water (LC-MS grade methanol, Fisher Scientific). The tissue–methanol mixture was subjected to bead-beating for 45 seconds using a Tissuelyser cell disrupter (Qiagen). Extracts were centrifuged at 5,000 rpm for 5 min to pellet insoluble protein and supernatants were transferred to clean tubes. The extraction procedure was repeated two additional times and all three supernatants were pooled, dried in a Vacufuge (Eppendorf) and stored at -80ºC until analysis. The methanol-insoluble protein pellet was solubilized in 0.2 M NaOH at 95ºC for 20 min and protein was quantified using a BioRad DC assay. On the day of metabolite analysis, dried cell extracts were reconstituted in 70% acetonitrile at a relative protein concentration of 3.1 µg/mL, and 4 µL of this reconstituted extract was injected for LC/MS-based targeted and untargeted metabolite profiling.

Plasma metabolites were extracted by addition of 1 part plasma to 20 parts 70% acetonitrile in ddH_2O (vol:vol). The mixture was briefly vortexed and then centrifuged at 16,000 ×g for 5 min to pellet precipitated proteins. An aliquot of the resulting extract (4 µL) was subjected to LC/MS untargeted metabolite profiling in positive and negative ion modes.

Urine metabolites were extracted by addition of 1 part urine to 10 parts 70% acetonitrile in ddH_2O (vol:vol). The mixture was briefly vortexed and then centrifuged at 16,000 ×g for 5 min. An aliquot of the supernatant (4 µL) was subjected to LC/MS untargeted metabolite profiling in positive and negative ion modes.

Metabolite extracts from tissue, plasma or urine were analyzed by LC/MS as described previously [9], using a platform comprised of an Agilent Model 1290 Infinity II liquid chromatography system coupled to an Agilent 6550 iFunnel time-of-flight MS analyzer. Chromatography of metabolites utilized aqueous normal phase (ANP) chromatography on a

Diamond Hydride column (Microsolv). Mobile phases consisted of (A) 50% isopropanol, containing 0.025% acetic acid, and (B) 90% acetonitrile containing 5 mM ammonium acetate. To eliminate the interference of metal ions with chromatographic peak integrity and electrospray ionization, EDTA was added to the mobile phase at a final concentration of 5 µM. The following gradient was applied: 0-1.0 min, 99% B; 1.0-15.0 min, to 20% B; 15.0 to 29.0, 0% B; 29.1 to 37min, 99% B. Raw data were analyzed using MassHunter Profinder 10.0 and MassProfiler Professional (MPP) 15.1 software (Agilent technologies). Student t-tests ($p < 0.05$) were performed to identify significant differences between groups.

To ascertain the identities of differentially expressed metabolites, LC/MS data was searched against an in-house annotated personal metabolite database created using MassHunter PCDL manager 8.0 (Agilent Technologies), based on monoisotopic neutral masses (<5 ppm mass accuracy) and chromatographic retention times. A molecular formula generator (MFG) algorithm in MPP was used to generate and score empirical molecular formulae, based on a weighted consideration of monoisotopic mass accuracy, isotope abundance ratios, and spacing between isotope peaks. A tentative compound ID was assigned when PCDL database and MFG scores concurred for a given candidate molecule. Tentatively assigned molecules were verified based on a match of LC retention times and/or MS/MS fragmentation spectra for pure molecule standards contained in a growing in-house metabolite database.

Isolation of primary proximal tubule cells from kidney cortices. Two fresh kidneys were harvested from newly sacrificed mice (WT and GCERA), cortices were collected and minced into small pieces on a tissue culture plate, and these were then transferred into 15-mL tubes containing 5 mL PBS and 1mg/mL collagenase IA (Millipore Sigma, C9891). The tubes were

placed on an Eppendorf Thermomixer R at 37 °C with gentle shaking for 30 minutes. Digestion was stopped with 5 mL DMEM containing 10% FBS. Each mixture was sieved through a 100 µm cell strainer (Falcon (Corning), 352360). Residual tissue particles were pressed with the sterile rubber plunger of a 3-mL syringe. Each tube was rinsed twice with 2 mL of DMEM, and each time the residual tissues were sieved through the 100 µm cell strainer. The filtrates were collected and sieved through a 70 µm cell strainer (Falcon (Corning), 352350). The samples were then washed once with 2 mL of DMEM. The filtrates were collected and then sieved through a 40 µm cell strainer (Falcon (Corning), 352340). The filtrates were centrifuged at 50 g at room temperature for 5 minutes. After discarding the supernatants, the pellets were resuspended in 15-20 mL complete Roswell Park Memorial Institute (RPMI) culture medium 1640 (Corning, 10-040-CM) supplemented with 10% fetal bovine serum, 20 ng/ml EGF (Peprotech, AF-100-15), insulin-transferrin-selenium-ethanolamine (ITS) (Gibco, 51500–056) and 1% penicillin-streptomycin in 15-cm cell culture plates. Cells were cultured in an incubator at 5% CO_2 and 37°C and the cells attached to the plate and grew. The next day, floating cells were removed, plates were rinsed with warm PBS, and fresh medium added.

Stable isotope tracing. When the cells became confluent in about 5-7 days, we seeded 0.5 x 10^6 – 0.6 x 10^6 cells per 3-cm plate. Forty-eight hours later, we washed cells with warm PBS twice, each time aspirating and removing as much PBS as possible. We then added 1 mL tracing medium (Gibco RPMI 1640 without glutamine, 21870-076) containing only 10% dialyzed FBS (Atlantis Bioscience, S181D-500), and 4 mM [$U^{13}C_5$]L-glutamine (MedChemExpress (MEC), HY-N0390S1) or 4 mM [^{12}C]L-glutamine, and incubated cells at 37 °C for either 2 or 24 hours. At 2 or 24 hours, we took cells out of the incubator, immediately removed medium, washed cells with cold PBS twice and with cold ddH_2O once, and aspirated and removed all residual PBS or

ddH_2O after each wash. We then immediately added 400 µL of 80% methanol prepared one day prior and kept at -75 °C overnight. The plates were placed on wet ice and cells scraped off the plates and transferred into a 2.0 mL special tube (Qiagen, 1050299) containing 1 grinding ball (OPS Diagnostics, GBSS 156-5000-01). The plates were rinsed with 150 µL cold 80% methanol twice, combining all 80% methanol mixtures in the same tube. The tubes were kept at -20°C for 10 min and metabolites were extracted or the samples were kept at -80°C and metabolites extracted later.

Metabolite extraction. The cell-MeOH mixture was beated for 45 seconds using the Tissuelyser II (Qiagen). The mixture was then kept at -20°C for 10 min, and centrifuged at 13.2 x 1000 rpm for 5 min. After Transferring the supernatants to new 1.5 mL Eppendorf tubes, 200 µL cold 80% MeOH was added to the pellets, and the above procedure was repeated twice. The supernatants were pooled and the pellets saved for protein quantification. A final centrifugation at the maximum speed for the pooled supernatants was performed and the final supernatants transferred to new 1.5 mL Eppendorf tubes. We combined any carry-over pellets with the pellets from the previous step. The combined pellets were dissolved in 150 µL 0.2 N NaOH for measurement of protein concentration by using the Pierce™ BCA Protein Assay Kit - Reducing Agent Compatible (Thermo Fisher, 23250).

The supernatants were dried by using a speed-vac (Savant) for about 4 hours until all MeOH and H_2O were removed. The samples were lyophilized and stored at -80°C. We then resolubilized the dried-down samples in 70% acetonitrile:30% H_2O by vortexing them on ice for 10 min; 3 cycles of vortexing and sitting on ice were performed. Then, we centrifuged samples at 20 x 1000 g, 4 °C, for 10 min, and transferred the supernatants to LC sample vials for

identification by LC/MS by utilizing an in-house untargeted, stable isotope tracing (USIT) workflow [9]. In this tracing experiment, we used proximal tubules from 4 WT mice and 3 ATF4 KO (GCERAΔ) mice. Under each condition (time and isotope), 1 sample from each mouse was run for LC/MS.

Assessment of renal functions. Renal functions were assessed by measuring serum blood urea nitrogen (BUN), creatinine, and albumin by the Laboratory of Comparative Pathology, Memorial Sloan Kettering, and by measuring urine creatinine and albumin by using the Quantichrome Creatinine assay kit (DICT-500) from BioAssay Systems, and the BCG Albumin Assay Kit (MAK124) from Sigma.

Data Processing. Principal component analysis (PCA) was performed with the R package clusterProfiler v4.6.2 [6], and the results were visualized using the R package ggplot2 v3.4.1 or by MetaboAnalyst 6.0. Volcano plots of $\log_{10}(p_{adj})$ versus $\log_2$(fold change) were generated by using GraphPad Prism 10.2.0 (GraphPad Software, San Diego, CA). The heatmap of the top 50 features for phenotypes was generated by Gene Set Enrichment Analysis (GSEA) of normalized gene expression. Pathways were generated by using Ingenuity Pathway Analysis (IPA) or MetaboAnalyst.

The statistical significance between groups was determined by unpaired two-tail t-test analysis ~~(black stars)~~, and p values of ≤ 0.05 were considered statistically significant.

References

1. Ebert SM, Dyle MC, Kunkel SD, Bullard SA, Bongers KS, Fox DK, Dierdorff JM, Foster ED, Adams CM (2012) Stress-induced skeletal muscle Gadd45a expression reprograms myonuclei and causes muscle atrophy. J Biol Chem 287: 27290-27301. DOI 10.1074/jbc.M112.374777
2. DiKun KM, Tang XH, Fu L, Choi ME, Lu C, Gudas LJ (2024) Retinoic acid receptor alpha activity in proximal tubules prevents kidney injury and fibrosis. Proc Natl Acad Sci U S A 121: e2311803121. DOI 10.1073/pnas.2311803121
3. Dobin A, Davis CA, Schlesinger F, Drenkow J, Zaleski C, Jha S, Batut P, Chaisson M, Gingeras TR (2013) STAR: ultrafast universal RNA-seq aligner. Bioinformatics 29: 15-21. DOI 10.1093/bioinformatics/bts635
4. Anders S, Pyl PT, Huber W (2015) HTSeq--a Python framework to work with high-throughput sequencing data. Bioinformatics 31: 166-169. DOI 10.1093/bioinformatics/btu638
5. Love MI, Huber W, Anders S (2014) Moderated estimation of fold change and dispersion for RNA-seq data with DESeq2. Genome Biol 15: 550. DOI 10.1186/s13059-014-0550-8
6. Wu T, Hu E, Xu S, Chen M, Guo P, Dai Z, Feng T, Zhou L, Tang W, Zhan L, et al. (2021) clusterProfiler 4.0: A universal enrichment tool for interpreting omics data. Innovation (Camb) 2: 100141. DOI 10.1016/j.xinn.2021.100141
7. Subramanian A, Tamayo P, Mootha VK, Mukherjee S, Ebert BL, Gillette MA, Paulovich A, Pomeroy SL, Golub TR, Lander ES, et al. (2005) Gene set enrichment analysis: a knowledge-based approach for interpreting genome-wide expression profiles. Proc Natl Acad Sci U S A 102: 15545-15550. DOI 10.1073/pnas.0506580102
8. Liberzon A, Birger C, Thorvaldsdottir H, Ghandi M, Mesirov JP, Tamayo P (2015) The Molecular Signatures Database (MSigDB) hallmark gene set collection. Cell Syst 1: 417-425. DOI 10.1016/j.cels.2015.12.004
9. Chen Q, Kirk K, Shurubor YI, Zhao D, Arreguin AJ, Shahi I, Valsecchi F, Primiano G, Calder EL, Carelli V, et al. (2018) Rewiring of Glutamine Metabolism Is a Bioenergetic Adaptation of Human Cells with Mitochondrial DNA Mutations. Cell Metab 27: 1007-1025 e1005. DOI 10.1016/j.cmet.2018.03.002

Supplementary Table 1 Primers for genotyping

Gene	Forward Primer	Reverse Primer
**Ggt CreEr**	5'GTTTCAATACCGGAGATCATGCAAG 3'	5' CAAGGCAGGGCTATTCTTCTTAGTG 3'
**AFT4**	5' GCCGGTTTAAGTTGTGTGCT 3'	5' CGTTCCTGCCTACATTGCTC3'
**Excised allele**	5'GCAGACGTTCCTGGGTTAGATACAATAAC 3'	5' GCCACTGTTTACTATCACCCCAGCC 3'
**36B4**	5' AGAACAACCCAGCTCTGGAGAAA 3'	5' ACACCCTCCAGAAAGCGAGAGT 3'

Supplementary Table 2

Sample	Barcode sequence	PF Clusters	% of the lane	% Perfect barcode	% One mismatch barcode	Yield (Mbases)	% PF Clusters	% >= Q30 bases	Mean Quality Score
EDM247	GAGAATGGTT+TTGCTGCCGA	24,533,428	1.06	98.70	1.30	4,956	100.00	96.18	36.37
EDM248	AGAGGCAACC+CCATCATTAG	20,347,005	0.88	98.54	1.46	4,110	100.00	96.19	36.37
EDM249	CCATCATTAG+AGAGGCAACC	19,647,599	0.85	98.34	1.66	3,969	100.00	95.92	36.34
EDM250	GATAGGCCGA+GCCATGTGCG	21,565,763	0.93	98.84	1.16	4,356	100.00	95.03	36.19
EDM251	ATGGTTGACT+AGGACAGGCC	23,413,251	1.01	96.16	3.84	4,729	100.00	95.75	36.30
EDM252	TATTGCGCTC+CCTAACACAG	22,489,447	0.97	98.63	1.37	4,543	100.00	96.22	36.38
EDM247	GAGAATGGTT+TTGCTGCCGA	23,654,278	1.01	98.85	1.15	4,778	100.00	96.46	36.42
EDM248	AGAGGCAACC+CCATCATTAG	19,405,974	0.83	98.67	1.33	3,920	100.00	96.47	36.42
EDM249	CCATCATTAG+AGAGGCAACC	18,737,207	0.80	98.50	1.50	3,785	100.00	96.14	36.38
EDM250	GATAGGCCGA+GCCATGTGCG	21,049,662	0.90	98.97	1.03	4,252	100.00	95.14	36.22
EDM251	ATGGTTGACT+AGGACAGGCC	22,385,115	0.95	96.59	3.41	4,522	100.00	95.99	36.35
EDM252	TATTGCGCTC+CCTAACACAG	21,837,295	0.93	98.72	1.28	4,411	100.00	96.49	36.43
EDM247a	AACCATAGAA+CCATCTCGCC	20,083,923	0.70	97.94	2.06	2,049	100.00	93.68	35.93
EDM252a	GGTTGCGAGG+TTGCTCTATT	20,144,429	0.70	96.38	3.62	2,055	100.00	93.16	35.83
JC031	GCCGCACTCT+CGAGGTCGGA	19,619,006	0.68	98.27	1.73	2,001	100.00	93.97	35.98
JC032	CCACCAGGCA+ATTCCATAAG	20,030,960	0.69	94.65	5.35	2,043	100.00	93.97	35.98
EDM247a	AACCATAGAA+CCATCTCGCC	20,283,789	0.70	98.03	1.97	2,069	100.00	93.82	35.95
EDM252a	GGTTGCGAGG+TTGCTCTATT	20,395,916	0.71	96.65	3.35	2,080	100.00	93.26	35.85
JC031	GCCGCACTCT+CGAGGTCGGA	19,827,480	0.69	98.38	1.62	2,022	100.00	94.09	36.01
JC032	CCACCAGGCA+ATTCCATAAG	20,214,671	0.70	95.19	4.81	2,062	100.00	94.10	36.01

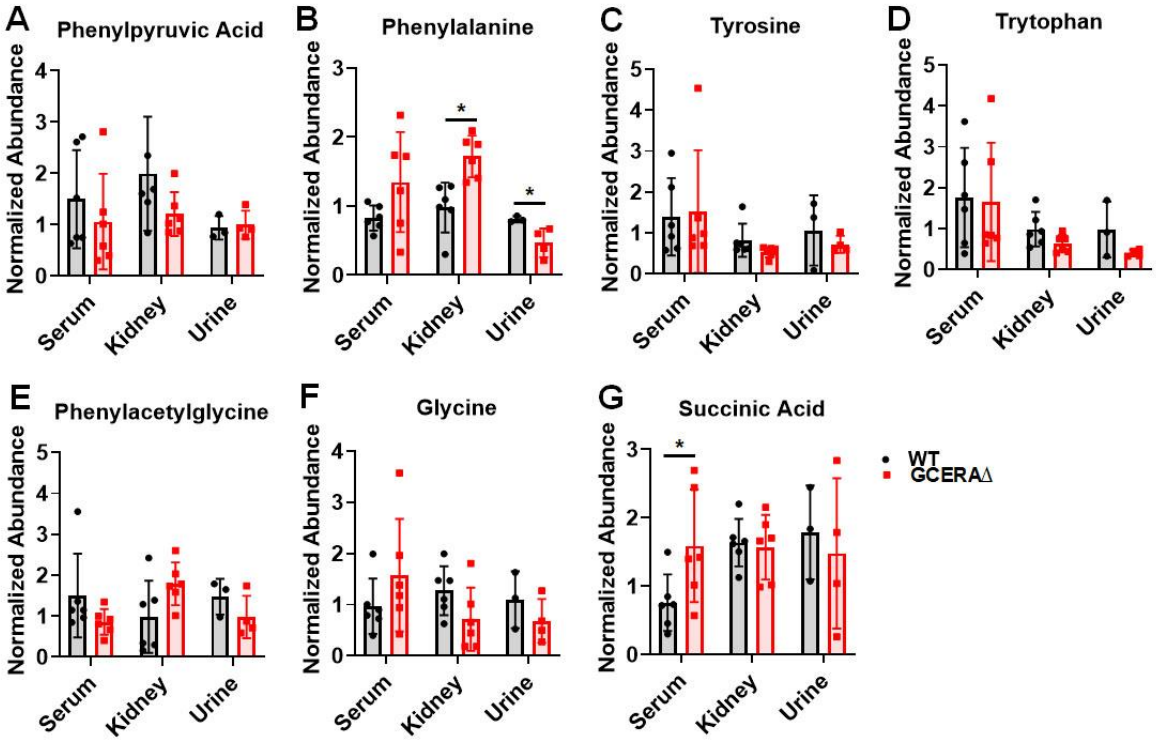

Supplementary Fig. 1. Effects of ATF4 deletion in proximal tubules on metabolites. Metabolites in serum, kidney and urine were obtained by metabolomics study. *$P \leq 0.05$.

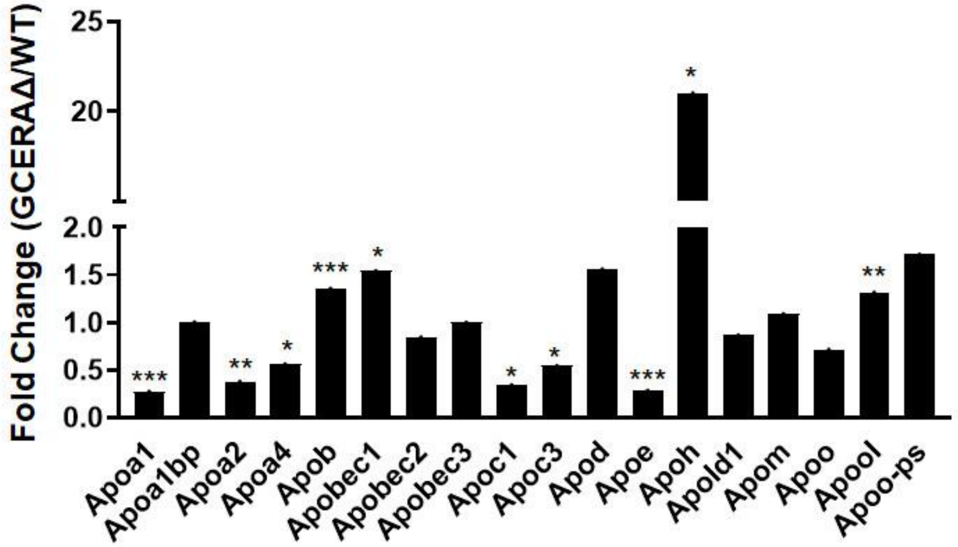

Supplementary Fig. 2. Effects of ATF4 deletion in proximal tubules on mRNAs of apolipoproteins. $^{*}P \leq 0.05$; $^{**}P \leq 0.01$; $^{***}P \leq 0.0001$.

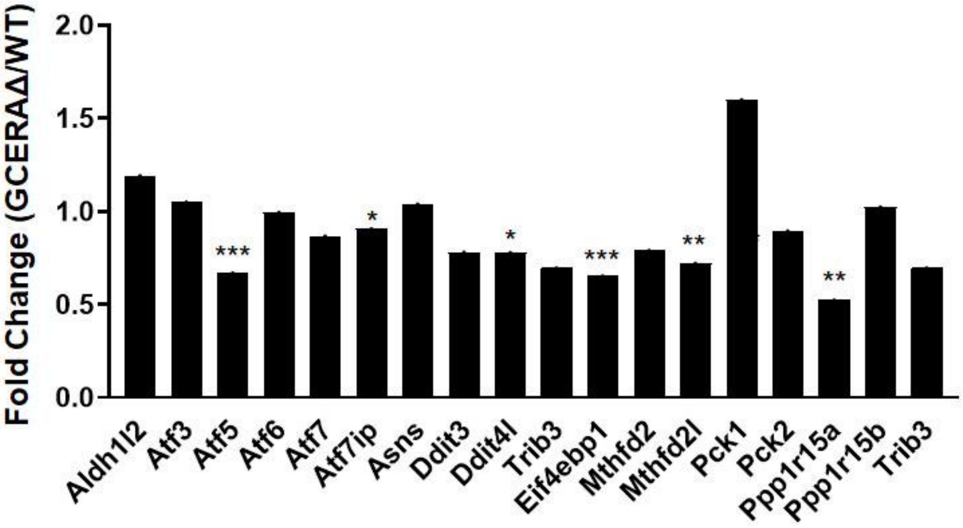

Supplementary Fig. 3. Effects of ATF4 deletion in proximal tubules on mRNAs of its target genes. *$P \leq 0.05$; **$P \leq 0.01$; ***$P \leq 0.0001$.
